# Three Cases of Chemical Burns Caused Due to Dimethyl Sulfate Poisoning

**DOI:** 10.7759/cureus.57060

**Published:** 2024-03-27

**Authors:** Shiori Mizuta, Tatsuya Miyakoshi, Kotaro Matsui, Yuko Nakayama, Yosinao Koshida

**Affiliations:** 1 Intensive Care Medicine, Toyama Prefectural Central Hospital, Toyama, JPN; 2 Emergency Medicine, Toyama Prefectural Central Hospital, Toyama, JPN

**Keywords:** dimethyl sulfate, poisoning, chemical burns, airway emergency, mass outbreak

## Abstract

Dimethyl sulfate (DMS) is a drug widely used as a pharmaceutical and synthetic raw material. On the other hand, it is highly toxic and requires management and treatment as a hazardous substance.

A mass outbreak of chemical burns resulting from DMS poisoning occurred at a drug factory. All three patients were brought to our hospital, a tertiary emergency medical facility, several hours after exposure. Their vital signs were stable, with only eye pain and a sore throat. However, after admission, two patients required emergency tracheostomy or endotracheal intubation due to laryngeal edema. Improvement was achieved through the administration of steroids, but a severely injured patient required an extended treatment period.

DMS poisoning is rare; however, it can be fatal depending on the exposure concentration. Furthermore, even if the initial symptoms are mild, laryngeal edema may develop later, requiring careful monitoring and appropriate airway interventions.

## Introduction

Dimethyl sulfate (DMS) is an alkylating agent widely used as a pharmaceutical and synthetic raw material. It is a clear, colorless, odorless, or slight odor, oily liquid that is absorbed through the mucous membranes or skin by inhalation of vapors through the skin or by ingestion [1,2]. The irritating properties of sulfuric acid and methyl hydrogen sulfate produced during DMS hydrolysis have corrosive effects on the eyes, respiratory tract, and skin. Methanol, which is also produced during DMS hydrolysis, can be absorbed throughout the body and cause convulsions and coma [3]. There is usually a delay of several hours between DMS exposure and symptom onset [4]. Strict care must be taken when handling it, as there is a possibility of fatal exposure without awareness.

Herein, we present cases of three patients who suffered from chemical burns caused by DMS poisoning in a drug factory. Reports of DMS poisoning are rare worldwide, with only one case reported in Japan [5,6]. We summarize the clinical condition and progress of the three patients we encountered and report on the basic knowledge of DMS along with a literature review.

The abstract of this article was previously presented at the 50th Annual Meeting of the Japanese Society of Intensive Care Medicine, held on March 2, 2023.

## Case presentation

This accident occurred in June 2023. First, the patient in Case 1 was transported to the emergency room, and at about the same time, the patient in Case 2 was also transported. The next day, the patient in Case 3 was referred to the ophthalmology outpatient department.

Case 1

The patient was a 44-year-old male with a history of depression and epilepsy. He worked with DMS and other materials to create the active ingredient for a specific drug at a pharmaceutical company’s drug factory. On the day of exposure, it was early summer, with the outdoor temperature of 28 °C [7] and the indoor temperature estimated to be over 30 °C. The workers wore helmets, goggles, masks for acid gas, latex gloves, and work clothes as protective equipment; however, they did not wear this equipment enough from the middle of the day. Approximately 5.5 h after exposure, the patient visited a nearby hospital with eye pain, lacrimation, nasal discharge, and a sore throat. Subsequently, he was referred to our hospital for the treatment of chemical burns.

**Figure 1:**
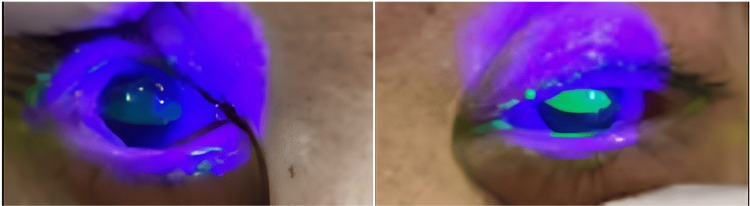
Corneal epithelial defects detected using corneal staining

**Figure 2:**
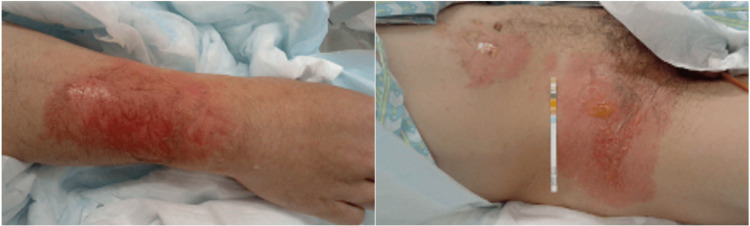
Burns on the right forearm, abdomen and groin

**Figure 3:**
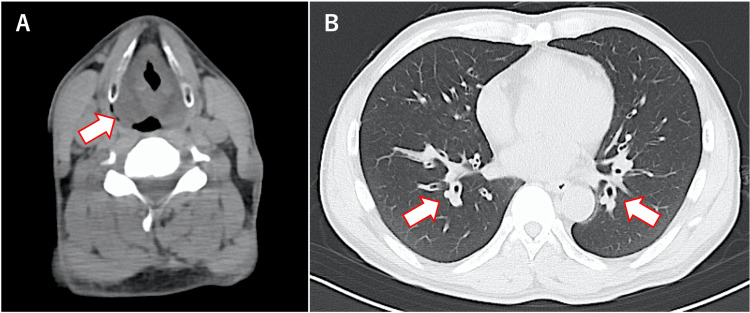
Computed tomography image showing laryngeal edema (A) and diffuse thickening of the bronchial wall (B)

At the time of admission, oxygen saturation (SpO_2_) was maintained at 97% on room air, and there was non-labored breathing; however, noticeable hoarseness was noted. No rales were heard in the chest. Palpebral conjunctival congestion and corneal epithelial defect were observed (Figure 1). In addition, burns were observed on the right forearm, abdomen, and right groin (I-IId burns, with an area of approximately 3%) (Figure 2). A blood test showed a high white blood cell (WBC) count (25,200 cells/μL). Computed tomography (CT) of the neck and chest revealed severe edema-like swelling from the nasal cavity to the larynx and diffuse thickening of the trachea and bronchial walls (Figures 3A, 3B). Based on the CT results, we were concerned about the risk of upper airway obstruction and attempted orotracheal intubation; however, the procedure was difficult because of significant laryngeal edema. Therefore, we prepared for surgical airway management, performed an emergency tracheostomy, and initiated ventilator management in the Intensive Care Unit (ICU).

**Figure 4:**
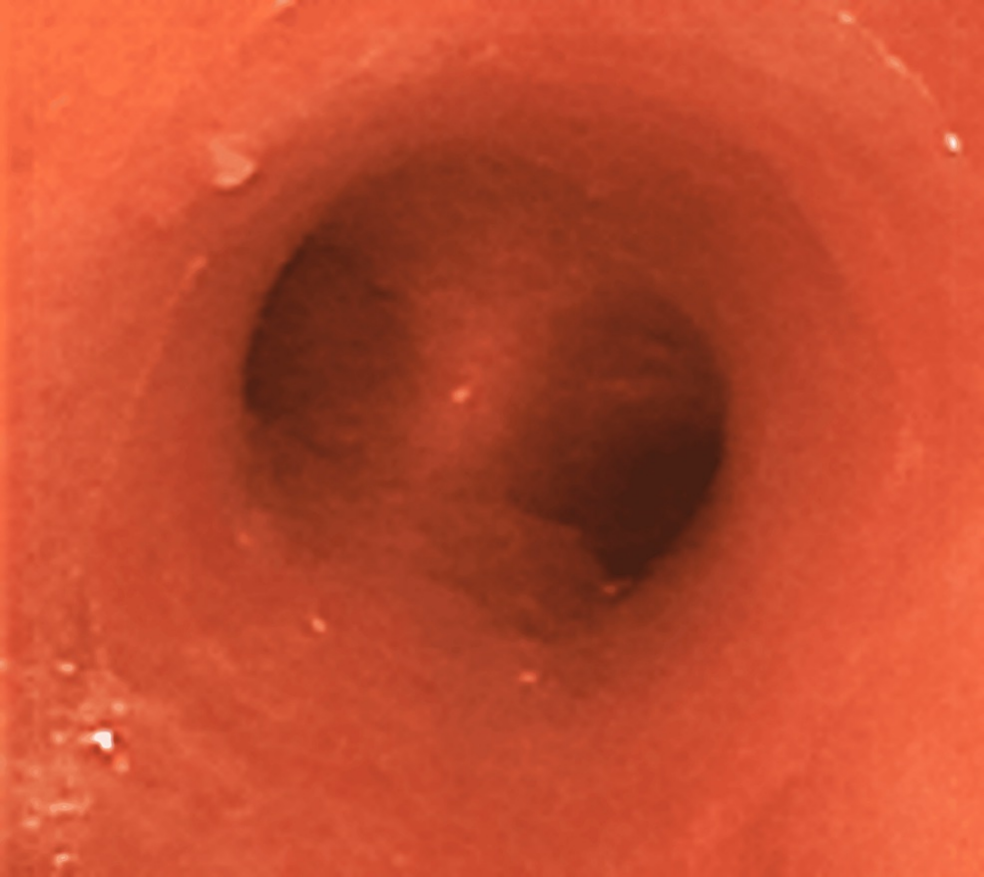
Redness of the bronchial mucosa

Intravenous corticosteroids (hydrocortisone (HDC) 300 mg, followed by dexamethasone 6.6 mg every 12 h) were started to treat the laryngeal edema. Bronchoscopy revealed redness of the mucous membrane extending from the trachea to the main bronchus. On the third day of hospitalization, the redness of the bronchial mucosa worsened and sputum production increased (Figure 4). Therefore, inhalation of unfractionated heparin (UFH) (5,000 units every 8 h) and acetylcysteine (352.4 mg every 8 h) was initiated. On the fourth day, the redness of the tracheal mucosa improved and sputum production started decreasing. Consequently, the patient was successfully weaned off the ventilator and supplied oxygen through a heat and moisture exchanger, and UFH was discontinued. He was transferred to the general ward on the fifth day, and the tracheostomy tube was replaced for the first time on the eighth day.

**Figure 5:**
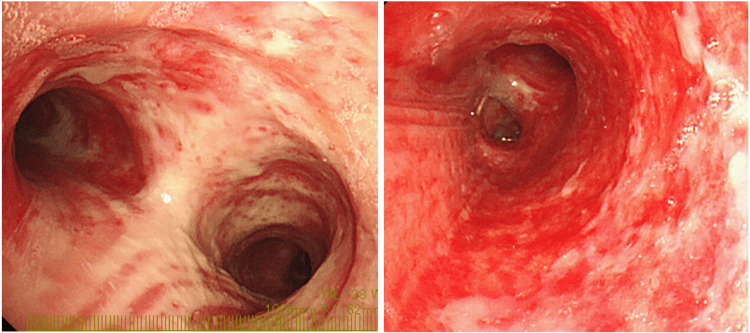
Severe inflammation of the bronchial mucosa

On the ninth day, he suddenly experienced decreased oxygenation and loss of consciousness, prompting the urgent replacement of the tracheostomy tube. Due to the expulsion of a significant amount of hard white sputum, it was assumed that the patient had temporarily suffocated. After readmission to the ICU, a heated nebulizer device was placed on the patient to enhance humidification, and he was closely monitored. Bronchoscopy revealed that the airway mucosa had turned white, indicating severe inflammation (Figure 5). As pneumonia was suspected, meropenem (1 g every 8 h) was administered intravenously until the 13th day. On the 14th day, the amount of sputum had decreased, and laryngeal edema had improved. Consequently, steroids were tapered and discontinued, and the tracheostomy tube was removed. Even after being transferred to the general ward on the same day, his respiratory condition remained stable.

Bronchoscopy on the 22nd day revealed that the airway mucosa was almost normal, and the probability of future scarring and stenosis was low. Corneal damage tended to heal with the use of steroids and antibiotic eye drops, and skin burns tended to heal with steroids and trafermin spray. He was discharged home on the 23rd day, followed up at the outpatient department, and completed treatment approximately nine months later.

Case 2

**Figure 6:**
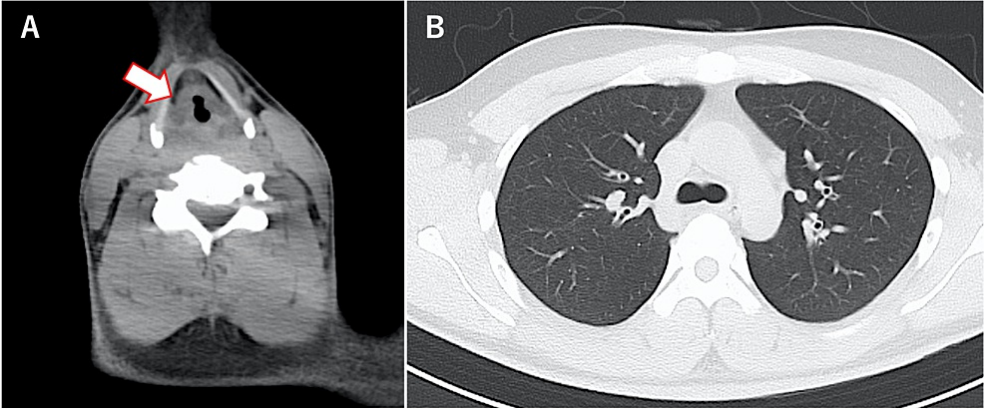
CT image showing mild edema over the vocal cords (A) and normal bronchial walls (B)

The patient was a 28-year-old male with no chronic illness. Similar to the patient described in Case 1, this patient worked directly with DMS while wearing protective equipment. Approximately 5.5 h after exposure, he presented to a nearby hospital with eye pain, lacrimation, nasal discharge, and sore throat and was referred to our hospital. On admission, SpO_2_ was maintained at 97% on room air; however, he had mild hoarseness. The patient also had palpebral conjunctival hyperemia and corneal epithelial defect. Blood tests showed a slight increase in WBC count (11,000/μL), and a CT scan of the neck and chest revealed mild swelling localized above the vocal cords, with no abnormalities detected in the trachea and lungs (Figure 6). After admission to the ICU, steroids (at the same dose as for the patient described in Case 1) were initiated. During monitoring, his hoarseness worsened approximately 10 h after exposure, necessitating orotracheal intubation. On the seventh day, the laryngeal edema improved, leading to the discontinuation of steroids and subsequent extubation. The patient was discharged on the 11th day.

Case 3

The patient was a 37-year-old male with no chronic illness. He did not directly use the DMS but occasionally visited the sites where patients described in Cases 1 and 2 worked. More than 10 hours after exposure, he experienced eye pain, lacrimation, nasal discharge, and throat discomfort. The following day, he visited a nearby doctor and was referred to the ophthalmology outpatient department of our hospital. SpO_2_ was 96% in room air, there was no hoarseness, blood tests showed a normal WBC level of 6,400/μL, and laryngoscopy did not reveal any edema. He was admitted to the emergency care unit for observation, and steroids (at the same dose as that for the patient described in Case 1) were started. Thereafter, no worsening of symptoms or laryngeal edema was observed, and the patient was discharged on the third day.

## Discussion

DMS poisoning is extremely rare in Japan, with only four cases being reported by administrative agencies [8] and one case reported in a previous study [5,6]. Although there have been reports of overseas outbreaks [9], this was the first reported case in Japan.

This case occurred during work involving the measurement of liquid DMS and powdered Sodium tert-Butoxide. According to interviews with drug companies and workers, these drugs did not come into direct contact with the worker's eyes, respiratory tract, or skin. The poisoning was determined to be caused by DMS, which can vaporize at 20 °C and rapidly reach harmful concentrations [1]. Since the patients did not exhibit any neurological symptoms such as headaches or disturbances of consciousness, and there were no laboratory findings indicating acidosis or an enlarged anion gap, no further tests or treatments for methanol poisoning were conducted. It is thought that working in a hot environment and not wearing enough protective equipment led to the exposure. This factory only handled DMS during the cold season, so it is possible that they were not careful enough. Previous reports have all occurred during periods of high temperatures [5,6,9]. This suggests that special care is required when handling DMS in hot weather.

Symptoms of DMS poisoning first appear in the eyes and then progress to the nose and upper respiratory tract. In addition, the greater the degree of exposure to DMS, the sooner the symptoms of poisoning appear; the more severe the symptoms, the longer they last [9]. The severity classification of the poisoning is as follows: (1) mild disease with congestion of the throat and larynx and airway erosion; (2) moderate disease with findings such as necrosis and detachment of the airway mucosa, as well as laboratory findings such as pneumonia, leukocytosis, and electrocardiogram changes; and (3) severe cases that may include laryngeal edema, non-cardiogenic pulmonary edema, myocardial injury, and encephalopathy [10]. In this study, patients described in Cases 1 and 2 exhibited severe symptoms, whereas those described in Case 3 exhibited mild symptoms. In Case 1, the individual most exposed to DMS developed symptoms earlier, experienced more severe symptoms, and had a longer hospitalization period. In all three cases, eye symptoms initially appeared; however, the patients self-diagnosed that there were no problems and simply observed their condition without cleaning their eyes. Protective equipment should be worn when working with DMS. It is important to understand that ocular symptoms are the initial indications of DMS poisoning. Prompt medical attention should be sought if symptoms occur, even if they are minor.

Early administration of high-dose corticosteroids is beneficial for the treatment of DMS poisoning [10,11]. This treatment reduces the incidence and severity of laryngeal and non-cardiogenic pulmonary edema and minimizes subsequent complications [10]. In addition, the effectiveness of prolonged erythromycin administration has been investigated in cases in which chronic lung damage leads to severe respiratory failure [6]. In our case, 300 mg HDC was administered during an early outpatient visit. Active steroid administration may prevent disease worsening.

Furthermore, in Case 1, UFH was administered via inhalation during the acute phase. Heparin inhalation is recommended in Japan as the initial treatment for airway injury because it prevents the progression of peripheral airway obstruction and lung injury caused by increased vascular permeability of the airway mucosa due to inflammation and the formation of a pseudomembrane [12]. In this case, the bronchoscopic findings improved, and the treatment was completed after two days. However, the patient’s condition suddenly worsened due to tube occlusion. Histopathological examination revealed that the aspirated solid sputum was a pseudomembrane containing a solidified fibrin clot, squamous epithelium, and inflammatory cells. In cases of DMS poisoning, which is thought to have a long-term treatment course, adequate humidification should be provided and UFH should be administered over a sufficient period.

Several months after exposure, the patient described in Case 1 presented to our hospital’s neurology department with muscle weakness on the right side of his body. Although the cause is unclear, the impact of DMS poisoning cannot be denied. Currently, there are no similar reports to date; however, more cases are expected in the future.

## Conclusions

We presented three cases of chemical burns caused by DMS. In workplaces where DMS is handled, workers must be mindful of the possibility of unintentional mass exposure to DMS and must pay special attention to eye symptoms. Special care must be taken during the hot season when DMS poisoning is more likely to occur. As medical professionals, we should understand that DMS poisoning can lead to delayed airway emergencies. Careful monitoring, early administration of steroids, and timely airway intervention are necessary. Adequate humidification and heparin inhalation may be helpful for severely injured patients, and long-term monitoring is important.
